# Remote Tower Air Traffic Controller Multimodal Fatigue Detection

**DOI:** 10.3390/s26061856

**Published:** 2026-03-15

**Authors:** Weijun Pan, Dajiang Song, Ruihan Liang, Zirui Yin, Boyuan Han

**Affiliations:** 1 Caac Academy of Flight Technology and Safety, Civil Aviation Flight University of China, Guanghan 618307, China; wjpan@cafuc.edu.cn; 2 College of Air Traffic Management, Civil Aviation Flight University of China, Guanghan 618307, China; lrh27500@foxmail.com; 3 School of Transportation and Logistics, Southwest Jiaotong University, Chengdu 611756, China; yin@my.swjtu.edu.cn; 4 Suining Flight College, Civil Aviation Flight University of China, Guanghan 618307, China; hanregulu@gmail.com

**Keywords:** remote tower, fatigue detection, multimodal fusion, cost-sensitive learning, XGBoost, electrocardiogram (ECG), eye tracking

## Abstract

**Highlights:**

**What are the main findings?**

- A multimodal framework integrating ocular and cardiac signals showed superior overall performance for fatigue detection in remote tower operations compared with conventional baseline models.
- ECG/HRV and eye-tracking features played complementary roles in characterizing fatigue-related physiological and behavioral changes.

**What are the implications of the main findings?**

- The proposed strategy improved the detection of minority fatigue events under severe class imbalance.
- Personalized calibration helped mitigate cross-subject performance degradation and improved deployment readiness.

**Abstract:**

Remote tower (rTWR) operations are reshaping air traffic control but introduce significant human-factor risks, notably cognitive fatigue induced by prolonged screen-based visual surveillance. To mitigate these risks in a safety-critical domain where missed detections can be catastrophic, we propose a non-intrusive, multimodal fatigue detection framework fusing ocular and cardiac signals. A high-fidelity simulation study with 36 controllers was conducted to collect eye-tracking and electrocardiogram (ECG) data, from which a 12-dimensional feature vector—integrating gaze entropy and heart rate variability (HRV)—was extracted. Addressing the severe class imbalance and scarcity of fatigue samples in physiological data, we developed a cost-sensitive XGBoost classifier combining SMOTE oversampling with a dynamically weighted loss function. Experimental results show that the proposed framework performed well under mixed-subject evaluation and improved sensitivity to fatigue events. Although a marked performance drop was observed under LOSO evaluation, personalized calibration partially alleviated this limitation, indicating the potential of the framework for real-time fatigue monitoring in remote tower operations.

## 1. Introduction

With the digitalization of the aviation transportation system, the remote tower (rTWR) is gradually replacing the traditional tower model, becoming a new approach to air traffic control. Kearney and Li’s study highlights that remote towers have evolved from the conceptual phase to regulatory recognition and engineering implementation stages [1]. However, compared to traditional towers, the “out-of-the-window view” is replaced by screen-mediated multi-angle information acquisition in remote towers. Controllers, during long shifts, are required to perform high-load visual search, target tracking, and multi-source information integration. Fatigue accumulates subtly and progressively, affecting situational awareness (SA) and decision-making quality, thus increasing system risk [2,3,4]. Current fatigue detection research mainly focuses on traditional tower models, neglecting the specific challenges faced by remote tower controllers. To address this gap and enhance safety, this study proposes a multimodal fatigue detection framework based on the fusion of eye-tracking and ECG signals.

Regarding fatigue assessment, although subjective rating scales can reflect individual experience, they often suffer from delayed responsiveness, limited repeatability, and potential interference with ongoing tasks, which makes them unsuitable for the continuous and minimally intrusive monitoring required in rTWR settings. Consequently, research has increasingly shifted toward objective fatigue detection based on behavioral and physiological signals. Among these approaches, eye-tracking (ET) has been widely adopted for workload and fatigue assessment in aviation due to its non-intrusive nature and strong interpretability. Peißl et al. conducted a selective review on the use of eye-tracking in aviation, focusing on how metrics such as fixations, saccades, and gaze distribution reflect attention allocation and information-processing dynamics during piloting and air traffic control tasks [5]. The study emphasizes the effectiveness of eye-tracking features in workload and fatigue assessment, noting that different eye movement behaviors—such as frequent saccades and prolonged fixations—can indicate varying cognitive load levels. Moreover, the paper discusses how eye movement can provide real-time, non-intrusive insight into attention and task performance. Ziv’s study further highlights the application of eye-tracking to understand visual attention mechanisms in aviation contexts. It demonstrates that eye-tracking can reveal critical insights into task strategies, particularly under complex task conditions, and provides a framework for understanding how pilots allocate visual attention [6]. Di Stasi and Diaz-Piedra pointed out that eye-movement research has expanded beyond conventional scanning behavior to include complexity-related measures such as gaze entropy, which can reveal how attentional resources are organized under complex operational demands [7]. For example, Diaz-Piedra et al. demonstrated in fighter pilot experiments that increasing task complexity significantly elevates gaze entropy, indicating more dispersed visual search and higher workload [8]. Moreover, within remote tower and monitoring tasks, display modalities and information complexity can substantially influence search efficiency and task performance. Liang et al. reported that different display formats can alter participants’ information acquisition strategies and completion efficiency [9]. In cockpit and ATC human–computer interaction studies, Li et al. showed that different message types elicited distinct visual scan patterns, thereby affecting interaction efficiency [10], whereas John et al. revealed physiological correlates associated with workload variation in tracking and collision prediction tasks [11]. Evidence from simulation and human–machine studies further supports the utility of oculometric features, including the “gaze freezing” phenomenon during simulator-induced spatial disorientation events [12], eye-tracking parameter systems for cognitive load measurement [13], and workload/performance prediction in human–robot interaction tasks [14]. In simulated taxiing and automated monitoring scenarios, researchers have also reported differences in scanning behavior and monitoring strategies between experts and novices [15,16]. Nevertheless, single-modality eye-tracking approaches remain sensitive to illumination changes, head movements, eyewear, and inter-individual differences in visual strategy, and some behavioral indicators become more pronounced only in the middle-to-late stages of fatigue. For instance, Caffier et al. noted that although blink parameters are related to drowsiness, their stability and applicability should be evaluated under specific operational constraints [17].

Beyond overt visual–behavioral signals, electrocardiography (ECG) and heart rate variability (HRV) can reflect workload fluctuations and fatigue accumulation through autonomic nervous system regulation and have thus been extensively used for fatigue recognition and state monitoring. Mukherjee and colleagues studied HRV as a physiological indicator of mental effort and fatigue [18]. They found that HRV was sensitive to variations in mental effort and may provide useful information for fatigue assessment. Their work emphasizes the importance of HRV in assessing cognitive load during sustained mental tasks, which aligns with its use in aviation for monitoring pilot and air traffic controller fatigue. Charles and Nixon’s systematic review indicated that physiological indices such as heart rate and HRV provide relatively stable representations of mental workload and are promising candidates for online monitoring [19]. Hughes et al. conducted a meta-analysis to explore the relationship between cardiac measures, such as HRV, and cognitive workload [20]. The analysis suggested that HRV, together with other cardiac indicators, is associated with cognitive workload and may serve as a useful physiological marker in fatigue-related assessment. This supports its integration in multimodal fatigue detection systems, as proposed in this research, where HRV is combined with eye-tracking data to improve fatigue detection accuracy. In simulated flight tasks, Mansikka et al. found that the mean inter-beat interval could effectively differentiate workload levels and complement subjective assessment scales [21]. In addition, recent work has explored lightweight deep learning architectures that directly process raw ECG signals for real-time stress/state feedback, providing a practical pathway for wearable implementations. For example, Tzevelekakis et al. proposed a lightweight CNN framework to enable real-time stress-level feedback from ECG signals [22]. Despite these advantages, ECG/HRV features can be affected by emotional fluctuations, respiratory rhythms, and motion artifacts, and they do not directly capture task-relevant visual search strategies. Therefore, in visually dominated rTWR operations, complementary multimodal information remains necessary.

Because a single modality is insufficient to comprehensively characterize fatigue evolution, research has progressively moved toward multimodal fusion frameworks that jointly model overt behavioral changes and latent physiological regulation. Borghini et al. reported that integrating neurophysiological signals such as EEG and ECG enables a more comprehensive characterization of workload, fatigue, and drowsiness in piloting and driving tasks, thereby enhancing assessment robustness [23]. In realistically simulated ATC tasks, Dasari et al. found that ICA-derived EEG features effectively captured mental fatigue, effort, and workload variations [24], while Ahn et al. demonstrated that simultaneous acquisition of EEG, ECG, and fNIRS helps reveal multi-system coupling changes associated with fatigue induced by sleep deprivation [25]. In driver fatigue and drowsiness research, Barua et al. proposed an automatic detection framework that fuses EEG, EOG, and contextual information, further illustrating the advantage of multi-source fusion for complex state recognition [26]. Kästle et al. reported correlations between EEG signals and SA, supporting objective SA characterization [27]. Li et al. further showed that combining EEG and eye-tracking features can discriminate SA differences under varying workload conditions in ATC tasks [28]. In adverse-weather shared SA detection, Yiu et al. incorporated EEG-enabled Bayesian neural networks, highlighting the potential of fusion modeling for understanding complex operational situations [29]. Meanwhile, Li et al. suggested that AI-enabled non-intrusive vigilance assessments could reduce a controller’s human error risk [30]. Rim et al. emphasized that deep learning on physiological signals is an emerging direction, although generalization and interpretability remain major concerns [31]. In engineering-oriented monitoring, Mou et al. proposed attention-based multimodal fusion for driver stress detection [32], and Martins et al. reviewed wearable fatigue monitoring technologies, noting both their potential and practical constraints [33].

Notably, research on fatigue monitoring has increasingly targeted rTWR-specific contexts. Liang et al. proposed a multimodal physiological data-driven approach for modeling visual fatigue in remote tower controllers by integrating eye-tracking, EEG, ECG, and EDA signals, and validated that fatigue induces cross-system synergistic changes in a simulated rTWR task [34]. Yin et al. introduced RTFnet, a multimodal fusion framework for fatigue detection in remote tower controllers, promoting the transition of rTWR fatigue monitoring toward real-time and engineering-oriented applications [35]. Furthermore, real-world driving datasets also indicate that fatigue/drowsiness samples are scarce and highly imbalanced. Martensson et al. achieved a drowsiness classification using physiological and driving performance data collected under real-road conditions, providing empirical references for model evaluation in realistic environments [36]. Overall, although multimodal fusion can improve recognition performance, rTWR datasets are typically characterized by limited sample sizes, strong noise, and pronounced inter-individual variability, making it essential to balance robustness and deployability.

In addition to model design, fatigue detection commonly faces the challenge of class imbalance and asymmetric safety costs: alert samples dominate, whereas fatigue samples are relatively scarce. If overall accuracy is overemphasized, models tend to favor the majority class and suffer from fatigue misses, which is particularly undesirable in safety-critical contexts. Krawczyk highlighted that learning from imbalanced data remains an open challenge and requires coordinated strategies such as resampling, cost-sensitive learning, and appropriate evaluation metrics [37]. Iosifidis et al. proposed AdaCC, demonstrating that introducing cumulative cost mechanisms into boosting frameworks can improve imbalanced classification performance [38]. Moreover, for structured small-sample datasets, tree-based and ensemble methods have shown good performance in engineering prediction tasks. Wu et al. further showed that ensemble learning can improve fatigue detection performance under imbalanced sample conditions and reduce miss rates for fatigue events [39]. Wang et al. reported that optimized XGBoost improves data-driven predictive accuracy, suggesting that lightweight ensemble models offer practical feasibility under limited data conditions [40]. While recent studies have explored multimodal fusion for rTWR fatigue, they frequently optimize for overall accuracy, which can mask the poor detection of rare fatigue events. The unique motivation of this study is to bridge the gap between physiological monitoring and the asymmetric safety constraints of ATC. Methodologically, we introduce a hybrid cost-sensitive learning framework that specifically penalizes missed fatigue detections, distinguishing our approach from conventional classifiers. The main contributions of this study are summarized as follows:

- Eye-tracking and ECG/HRV signals are collected from 36 air traffic controllers in a high-fidelity remote tower simulation, providing a multimodal dataset for fatigue modeling in rTWR operations.
- A feature-level fusion framework based on cost-sensitive XGBoost was developed to address severe class imbalance by integrating SMOTE and dynamically weighted loss.
- A personalized calibration strategy was designed to mitigate cross-subject generalization loss, and feature importance analysis was conducted to interpret the relative contributions of multimodal features to fatigue detection.

## 2. Experiments

### 2.1. Design

The experiment was conducted under a simulated remote tower setting, and all task scenarios were designed under uniform meteorological conditions to ensure comparability across conditions.

To reflect typical operational demands in remote tower operations, three representative task scenarios were designed, covering routine operations, high-traffic operations, and abnormal event handling:

- Scenario 1 (Routine operation): Three arriving and four departing aircraft, with no more than two aircraft on taxiways at any time, resulted in low traffic pressure. The task difficulty was moderate, closely resembling routine remote tower operations.
- Scenario 2 (High-traffic operation): Eight arriving and eight departing aircraft, with up to five aircraft taxiing simultaneously during peak periods, significantly increased surface traffic complexity and coordination demands. This scenario mimicked long-term traffic growth and workload escalation during sustained remote tower deployment.
- Scenario 3 (Abnormal event handling): The traffic volume was identical to Scenario 1, but one aircraft experienced a technical malfunction, causing congestion. Controllers needed to adjust taxi routes to prevent conflicts and ensure sufficient resources for towing the disabled aircraft. This scenario was presented less frequently to maintain realistic distributions and ensure data adequacy for analysis.

Prior to the formal experiment, all participants received a detailed briefing on the experimental procedure, completed an adaptation/practice session, and provided written informed consent. In the 5-min period immediately before the start of each experimental set, physiological acquisition devices were calibrated and signal quality was verified; data collected during this period were excluded from subsequent analyses.

During the experiment, participants maintained a standard seated air traffic control posture. The simulation environment consisted of three synchronized displays, presenting a panoramic out-the-window view that included aircraft, runway, taxiway, and apron/stand areas. To enhance ecological validity, one researcher acted as the pilot in a separate isolated room and interacted with the controller in real time via a voice communication system, thereby approximating realistic radiotelephony communication.

A within-subject design was adopted. Each participant completed two experimental sets, comprising a total of 10 test scenarios (four Scenario 1 trials, four Scenario 2 trials, and two Scenario 3 trials). To mitigate order effects, flight sequences and call signs within each scenario type were randomly generated. The two experimental sets were scheduled on different days, starting at 09:30 and 14:30, respectively, with an inter-session interval of 24 h. A 5-min rest break was provided between consecutive scenarios, and the total duration of each set was approximately 1.6 h.

### 2.2. Experimental Protocol

This study employed the Tower Client simulation platform to establish a high-fidelity remote tower operational environment. The system was designed to closely replicate the human–machine interaction (HMI) logic and aircraft dynamic behaviors encountered in real-world air traffic control (ATC). Participants acquired real-time visual information of the apron, runway, and surrounding airfield area through a high-resolution panoramic wide-field-of-view (FOV) display system, thereby emulating the tower out-of-the-window (OTW) view.

**Figure 1:**
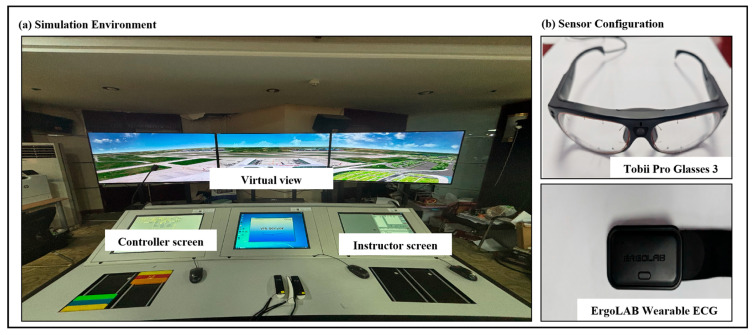
Experimental setup and data acquisition system for remote tower operations. (**a**) The high-fidelity remote tower simulation environment where participants perform simulated air traffic control tasks. (**b**) Configuration of multimodal physiological sensors, featuring the wearable eye tracker worn by the participant to capture gaze behavior, and the wearable ECG sensor attached for heart rate variability monitoring.

From an experimental control perspective, the simulator supports instructor-driven standardized test scenarios and integrates a real-time supervision and monitoring module, which enables synchronized visualization of both the controller-side and pilot-side operational states, as well as time-stamped action sequence logs. In terms of operational interaction, participants used a keyboard-and-mouse interface as the primary input modality to issue ATC commands, including pushback clearance, taxi route planning, and takeoff/landing clearance issuance. Figure 1 illustrates the experimental setup in detail.

During the experiment, three types of data were collected: (i) oculometric data acquired using Tobii Pro Glasses 3 wearable eye tracker at a sampling rate of 100 Hz; (ii) cardiac data for HRV analysis recorded using an ErgoLAB bio-sensing wearable ECG device at a sampling rate of 512 Hz; and (iii) subjective fatigue ratings assessed with the Samn–Perelli 7-point fatigue scale (SP-7) [41]. To preserve the continuity of the remote tower control task and avoid disrupting participants’ cognitive flow, participants completed the SP-7 scale (see Table 1) immediately before each scenario to establish a baseline state and immediately after the scenario to capture accumulated fatigue. The SP-7 score recorded at the end of each scenario was used as the ground-truth label for all signal segments extracted from that specific scenario. After preprocessing and removing segments containing substantial signal artifacts or missing data, a total of 2812 valid multimodal samples were retained for subsequent feature extraction and model training.

A total of 36 participants were recruited from the School of Air Traffic Management, Civil Aviation Flight University of China, including six senior ATC instructors and 30 trainees. All participants met CAAC Class I medical certification standards, had normal vision, and completed an adaptation session before the formal experiment. The study was approved by the CAFUC Ethics Committee, and written informed consent was obtained from all participants, who were informed of their right to withdraw at any time without penalty. All data were anonymized to protect participant confidentiality.

## 3. Methods

### 3.1. Data Segmentation and Sample Distribution

A sliding window segmentation approach with a window length of 60 s and a step size of 60 s was employed to extract both time-domain and frequency-domain features from continuous physiological recordings. This non-overlapping segmentation strategy was implemented to ensure statistical independence between samples and to mitigate data redundancy, thereby maintaining temporal separation between the training and test sets. Following segmentation and artifact rejection, a total of 2812 valid samples were retained, encompassing the full experimental recordings from 36 participants. To assess cross-subject balance, the sample contribution of each participant was quantified. The results revealed that, on average, each participant contributed 96 samples (median = 96, range = 88–102), with a low standard deviation of 3.5 samples. Given that the maximum deviation from the mean did not exceed 10%, the dataset showed relatively good inter-subject balance, negating the need for additional subject-level weighting or resampling strategies.

### 3.2. Labeling Strategy and Reliability Verification

#### 3.2.1. Extreme-Group Labeling Approach

After segmentation, each time window must be assigned a corresponding fatigue state label based on the Samn–Perelli 7-point fatigue scale (SP-7). To formulate a robust binary supervised learning task, we applied an extreme-group approach:

- Alert (Negative class): SP-7 scores 1–3 (Fully alert to Okay, somewhat fresh).
- Fatigue (Positive class): SP-7 scores 5–7 (Moderately tired to Completely exhausted).

Samples with SP-7 score 4 (A little tired) were excluded (18.5% of data). This exclusion was mainly based on two considerations: (1) operational decision-making in ATC requires relatively unambiguous binary judgments; and (2) the intermediate level shows greater subjective variability, which may blur the decision boundary. By focusing on more distinct physiological states, the model receives clearer supervisory signals, while its continuous probability output can still serve as a proxy for early warning monitoring.

Ultimately, this resulted in 2812 samples (2655 alert, 157 fatigue) with a class imbalance of 16.9:1.

#### 3.2.2. Physiological Validation of Labels

Although the SP-7 scale is a standardized subjective assessment tool, relying solely on self-reported ratings may introduce bias due to individual differences in fatigue perception. To rigorously validate the reliability of the ground-truth labels defined in Section 3.2.1, we incorporated PERCLOS (Percentage of Eyelid Closure) as an objective physiological benchmark. PERCLOS, widely recognized as a “gold standard” indicator for drowsiness detection, measures the proportion of time within a given interval during which the eyelid covers more than 80% of the pupil [42].

Using the cleaned dataset (i.e., after excluding SP-7 = 4 samples), we conducted validation at two levels:

- Correlation analysis: Spearman’s rank correlation analysis revealed a statistically significant positive correlation (r = 0.94, *p* < 0.001) between subjective SP-7 ratings and the corresponding PERCLOS values, indicating strong concordance between perceived fatigue and objective physiological drowsiness [43].
- Between-group difference testing: To verify the effectiveness of the extreme-group strategy, we compared the PERCLOS distributions between the defined Alert group (*n* = 2655) and Fatigue group (*n* = 157). An independent-samples *t*-test demonstrated a statistically significant difference between the two groups (t = −32.08, *p* < 0.001), with the Fatigue group exhibiting a higher proportion of eye closure.

The above results confirm that the binary labels generated in this study exhibit good construct validity, accurately capturing controllers’ underlying cognitive states and thereby supporting their appropriateness as ground-truth annotations for supervised learning models.

### 3.3. Data Preprocessing

Because raw physiological recordings are highly susceptible to contamination from environmental noise, participant motion artifacts, and instrumentation-related electrical interference, a systematic signal preprocessing pipeline was established to ensure the accuracy and reliability of subsequent feature extraction.

#### 3.3.1. Ocular Signal Denoising and Event Classification

The preprocessing of eye-tracking data aimed to correct blink-induced missing samples and transform continuous gaze trajectories into semantically meaningful visual events.

Blink Artifact Removal and linear interpolation: Blink episodes typically manifest as instantaneous drops in pupil diameter to zero. We first identify the time intervals where pupil diameter equals zero. For normal blinks (lasting between 75 and 500 ms), missing gaze-point coordinates were reconstructed using linear interpolation to maintain the temporal continuity of the time series. Similar interpolation-based approaches for reconstructing missing data have been reported in related studies [44].

**Figure 2:**
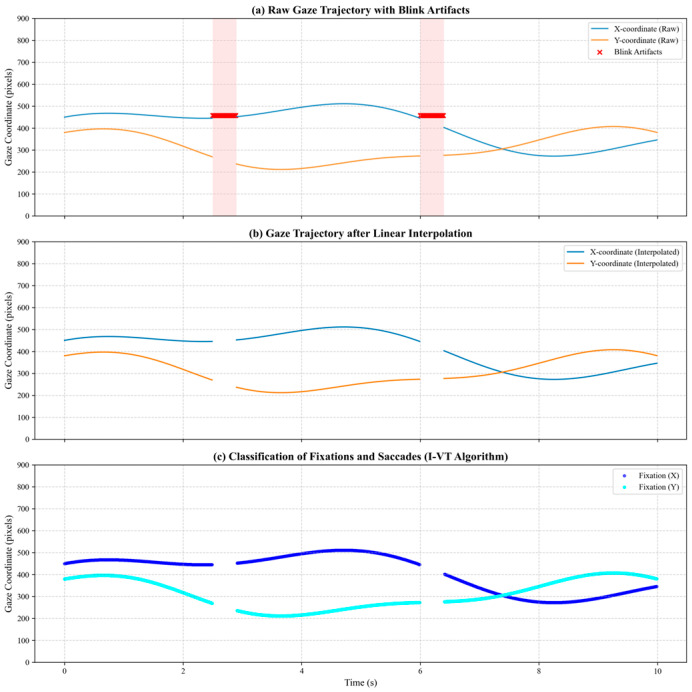
Schematic of ocular signal processing. (**a**) Raw gaze trajectory with blink artifacts; (**b**) reconstructed trajectory after interpolation; (**c**) fixation components extracted from the interpolated gaze trajectories using the I-VT algorithm.

Event Classification via I-VT Algorithm: To distinguish between fixations and saccades, this study employs the velocity threshold identification (I-VT) algorithm, which classifies eye movements by calculating the angular velocity of the gaze between adjacent sampling points. Specifically, when the angular velocity is below 30°/s, the gaze is classified as a fixation, indicating that the controller is engaged in information extraction; when the angular velocity is above or equal to 30°/s, it is classified as a saccade, indicating a rapid shift in gaze. This method has been widely adopted in similar research [45]. Figure 2 illustrates the ocular signal processing.

#### 3.3.2. Cardiac Signal Filtering and R-Peak Detection

For the original ECG signals sampled at 512 Hz, the preprocessing focus is on eliminating power-line interference and baseline drift, as well as accurately locating the QRS complex.

Wavelet-based signal enhancement is employed, utilizing Discrete Wavelet Transform (DWT) to remove noise. The Symlet (sym8) wavelet basis is selected for an 8-level decomposition [46]. To suppress noise while preserving the morphological features of the ECG signal, the high-frequency detail coefficients $d_{j,k}$ are processed using a soft thresholding function.

(1) $$\begin{array}{c} \begin{array}{c} {\hat{d}}_{j,k}=\left\{\begin{array}{ll} \mathrm{sgn}(d_{j,k})(|d_{j,k}|-\lambda ), & \;\;|d_{j,k}|\;\geq \;\lambda \\ 0, & \;\;|d_{j,k}|\;<\;\lambda \end{array}\right. \end{array} \end{array}$$

In Formula (3), λ is the universal threshold, defined as $\lambda =\sigma \sqrt{2\log N}$. The reconstructed signal effectively removes electromyographic noise and baseline drift.

Pan–Tompkins algorithm R-Peak Detection [47]: In this study, the Pan–Tompkins algorithm is used to detect R-peaks. The algorithm applies bandpass filtering, differentiation, and squared processing, followed by moving window integration, to obtain the waveform envelope, emphasizing the characteristics of the QRS complex. The integration function is defined as:

(2) $$\begin{array}{c} \begin{array}{c} y(n)=\frac{1}{W}\sum_{i=0}^{W\;-\;1}x^{2}(n\;-\;i) \end{array} \end{array}$$

where W is the window width (set to 150 ms in this study). Then, an adaptive thresholding strategy is applied to locate the local maxima in y(n), which corresponds to the R-wave peak moments ($T_{R}$). Finally, the R-R interval sequence (R-R intervals) is calculated as:

(3) $$\begin{array}{c} \begin{array}{c} {RRI}_{i}=T_{R_{i+1}}-T_{R_{i}} \end{array} \end{array}$$

**Figure 3:**
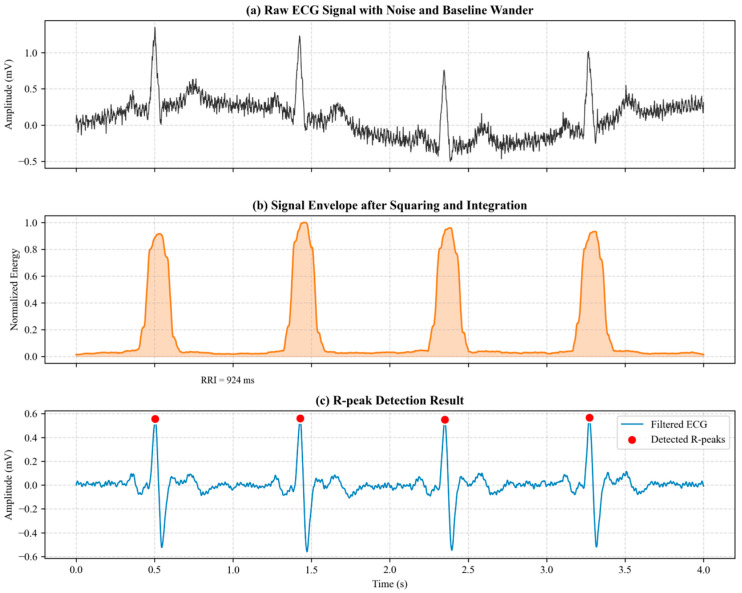
ECG signal processing pipeline. (**a**) Raw ECG signal with baseline wander; (**b**) signal envelope after moving window integration; (**c**) detected R-peaks (red dots) for HRV calculation.

Any R-R intervals deviating by more than ±20% from the local mean are identified as ectopic beats and removed. Figure 3 illustrates the ECG signal processing pipeline.

### 3.4. End-to-End Pipeline of the Proposed Method

**Figure 4:**
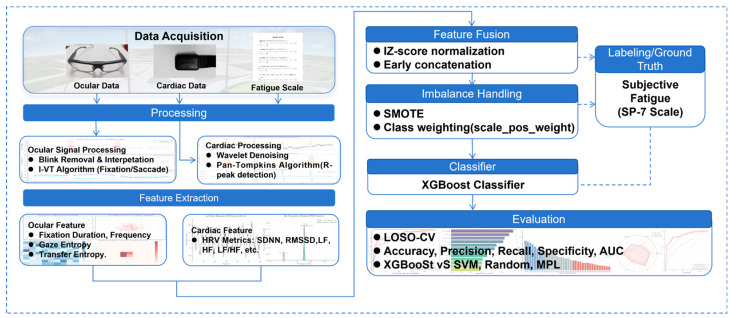
Technical roadmap of multimodal physiological feature extraction for fatigue detection.

Figure 4 summarizes the end-to-end technical roadmap from raw physiological signals to model evaluation. Ocular and cardiac signals are first processed in two independent pipelines: eye-tracking data are cleaned with blink handling/interpolation and event parsing, whereas ECG signals are denoised and used for R-Peak Detection. Based on the processed signals, modality-specific features are extracted, including oculometric features (e.g., fixation metrics and gaze entropy descriptors) and cardiac features derived from HRV analysis (e.g., SDNN and RMSSD, as well as frequency-domain indices). The extracted features are then fused by concatenation to form a unified multimodal feature vector. Ground-truth labels are assigned according to the fatigue rating protocol, and class imbalance is addressed before training the classifier. Finally, the trained model is evaluated under the predefined protocols (mixed-subject split and LOSO-CV), and performance is reported using standard metrics such as accuracy, precision, recall/sensitivity, specificity, and AUC.

### 3.5. Feature Extraction

After the aforementioned signal preprocessing, this study extracts multidimensional physiological features from both ocular and cardiac data to quantify the cognitive load and mental fatigue state of air traffic controllers. The feature extraction process aims to transform the raw, complex physiological signals into discriminative information that can be effectively recognized by machine learning models, thereby providing a basis for subsequent classification tasks.

#### 3.5.1. Ocular Behavior Features

Visual scanning is the primary method for remote tower controllers to gain situational awareness, and subtle changes in eye movements are linked to mental fatigue. This study extracts statistical and nonlinear dynamic features from eye-tracking data to quantify cognitive load and fatigue.

Key features include PERCLOS (Percentage of Eyelid Closure), a “gold standard” for fatigue detection, which identifies drowsiness through eyelid activity. Blink frequency (BF) and mean blink duration (MBD) are also calculated, as fatigue causes changes in blink frequency and slower blinks, reflecting reduced neuromuscular control.

Additionally, we focus on fixation behavior and pupil diameter. Longer mean fixation duration (MFD) and lower fixation frequency (FF) reflect decreased visual search efficiency with increasing fatigue. Pupil diameter variability (PDV) is also measured, as parasympathetic activation from fatigue causes pupillary instability, leading to higher PDV.

To capture fatigue-induced degradation in scanning strategy, we introduce two entropy-based indicators: Stationary Gaze Entropy (SGE) and Transition Gaze Entropy (TGE). These quantify the disorder in visual attention distribution and transitions, offering a comprehensive measure of fatigue’s impact on visual control.

**Figure 5:**
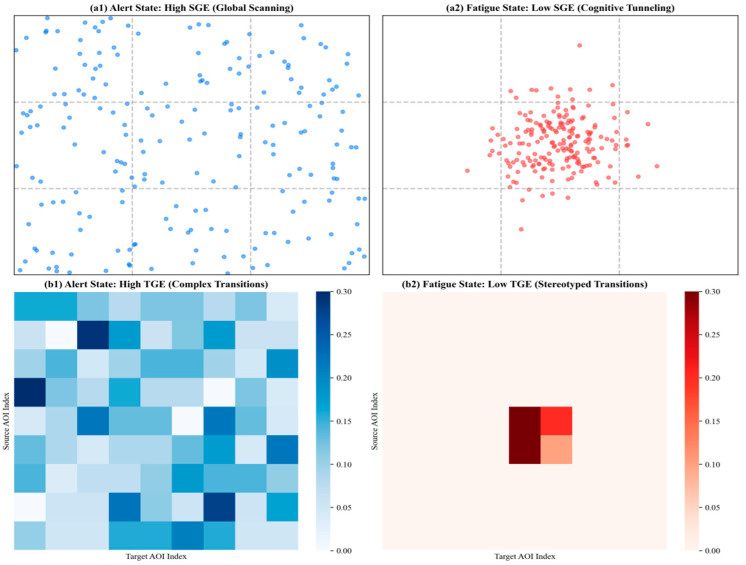
Illustration of visual entropy metrics under different cognitive states. Subfigures (**a1**) and (**a2**) show Stationary Gaze Entropy (SGE) patterns in the alert state (high SGE) and fatigued state (low SGE), respectively. Subfigures (**b1**) and (**b2**) show Transition Gaze Entropy (TGE) represented by transition probability matrices in the alert state (high TGE) and fatigued state (low TGE), respectively.

Stationary Gaze Entropy (SGE) is used to quantify the discreteness of fixation point distribution across the panoramic screen space. The remote tower’s panoramic display is virtually divided into an M × N grid of regions (AOIs). Let $p_{i}$ be the probability of a fixation point falling within the i-th grid, then SGE is defined as:

(4) $$\begin{array}{c} H_{\mathrm{SGE}}=\;-\sum_{i=1}^{K}p_{i}\log_{2}(p_{i}) \end{array}$$

where K is the total number of non-empty grids. Higher SGE values reflect broad scanning, while a reduction in SGE during fatigue indicates more focused, constrained attention, representing the “cognitive tunnel” effect (Figure 5a1,a2).

Transition Gaze Entropy (TGE) is used to quantify the regularity of gaze transitions between different areas of interest (AOIs). By constructing a transition probability matrix T, where the element $p_{\mathrm{ij}}$ represents the conditional probability of gaze transitioning directly from region i to region j, TGE is defined as:

(5) $$\begin{array}{c} H_{\mathrm{TGE}}=\;-\sum_{i=1}^{K}p_{i}\sum_{j=1}^{K}p_{\mathrm{ij}}\log_{2}(p_{\mathrm{ij}}) \end{array}$$

As shown in Figure 5b1,b2, higher TGE values indicate complex and non-repetitive scanning paths, while lower TGE values suggest that the scanning strategy has degraded into a stereotyped and predictive pattern, which is a significant indicator of visual search strategy failure under fatigue.

#### 3.5.2. Cardiac Physiological Features

HRV is a well-established indicator of autonomic nervous system (ANS) regulation of the heart. This study extracts time-domain and frequency-domain HRV features based on the R-R interval sequence detected by the Pan–Tompkins algorithm [47] to assess mental load and alertness.

In time-domain analysis, we calculate SDNN and RMSSD:

(6) $$\begin{array}{c} SDNN=\sqrt{\frac{1}{N-1}\sum_{i=1}^{N}(RR_{i}-\bar{\mathrm{RR}}{)}^{2}} \end{array}$$

(7) $$\begin{array}{c} RMSSD=\sqrt{\frac{1}{N-1}\sum_{i=1}^{N-1}(RR_{i+1}-RR_{i}{)}^{2}} \end{array}$$

**Figure 6:**
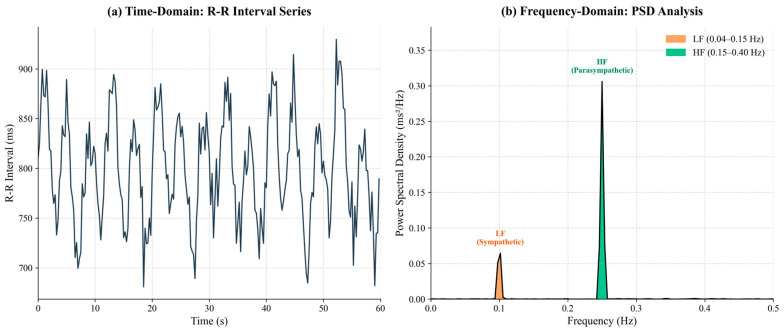
Time- and frequency-domain HRV analysis. (**a**) R-R interval series. (**b**) PSD spectrum showing LF (orange) and HF (green) components for calculating the sympathovagal balance ratio (LF/HF).

To reveal the balance of the autonomic nervous system (ANS), we used Fast Fourier Transform (FFT) [48] to calculate the power spectral density (PSD) of the R-R interval sequence, with a typical spectral distribution shown in Figure 6.

Low-frequency power (LF, 0.04–0.15 Hz) reflects both sympathetic and parasympathetic activity, but mainly sympathetic under high loads.

High-frequency power (HF, 0.15–0.40 Hz) reflects parasympathetic regulation, especially linked to respiratory sinus arrhythmia (RSA).

LF/HF ratio quantifies the sympathovagal balance, with a lower ratio typically indicating fatigue due to reduced sympathetic activity and increased parasympathetic activity.

### 3.6. Multimodal Fusion and Classification Model

#### 3.6.1. Feature Normalization and Fusion

Since eye-tracking features (e.g., pupil diameter, in mm) and cardiac features (e.g., LF/HF, dimensionless) have different physical dimensions and data ranges, directly inputting them into the model may lead to training instability. Therefore, prior to fusion, we first apply Z-score normalization [49] to each feature dimension:

(8) $$\begin{array}{c} x’=\frac{x-\mu }{\sigma } \end{array}$$

In Formula (9), μ and σ represent the mean and standard deviation of the respective feature dimension in the training set.

Subsequently, an early fusion strategy [50] is employed to construct a multimodal feature space. The standardized eye-tracking feature vector $V_{\mathrm{ocular}}$ (including PERCLOS, BF, MBD, MFD, FF, PDV, SGE, TGE) is serially concatenated with the cardiac feature vector $V_{\mathrm{cardiac}}$ (including MeanRR, SDNN, RMSSD, LF/HF) to generate a unified D-dimensional feature vector $V_{\mathrm{unified}}$ (where D = 12 in this study). This fused vector simultaneously represents the controller’s external behavioral performance and internal neural regulatory state.

#### 3.6.2. Hybrid Class Imbalance Handling Strategy

In real-world remote tower operations, the class imbalance between alert (negative) and fatigue (positive) samples is significant, with a ratio of approximately 1:16.9. Using the original data for training leads to a “majority class trap”, where the classifier predicts all samples as the negative class, resulting in a high false negative rate for fatigue detection.

To address this, we propose a hybrid strategy combining data-level augmentation and algorithm-level weighting. This approach enhances the model’s sensitivity to the minority class by improving both feature distribution and loss optimization. Specifically, we employ the Synthetic Minority Over-sampling Technique (SMOTE) [51], which generates synthetic samples through linear interpolation, increasing feature diversity and reducing overfitting risk compared to random oversampling.

Specifically, for each minority class (fatigue) sample $x_{i}$ in the training set, the algorithm first computes its k-nearest neighbors in the feature space (with k = 5 in this study). Then, a random sample x_k_ from the k-nearest neighbors is selected, and a new synthetic sample $x_{\mathrm{new}}$ is generated along the line connecting the two samples. The mathematical expression is as follows:

(9) $$\begin{array}{c} x_{new}=x_{i}+\delta \cdot ({\hat{x}}_{i}-x_{i}) \end{array}$$

In Formula (11), δ is a random variable uniformly distributed in the interval [0, 1]. Through this process, the decision boundary of fatigue samples is expanded, enabling the model to learn a more generalized feature representation.

During the training process of the XGBoost model [52], we restructured the binary classification log-loss function into a weighted form. To ensure the rigor and fairness of the experiment, the balanced weights are not set as a global fixed value, but are dynamically computed within each training fold during cross-validation.

For the k-th training fold $D_{\mathrm{train}}^{k}$, the positive class (fatigue) weight $w_{\mathrm{pos}}^{k}$ is defined as the ratio of the number of negative class samples to the number of positive class samples:

(10) $$\begin{array}{c} w_{pos}^{k}=\frac{N_{negative}^{k}}{N_{positive}^{k}} \end{array}$$

This weight is directly applied to the gradient update formula of the loss function:

(11) $$\begin{array}{c} g_{i}=\sigma ({\hat{y}}_{i})-y_{i}\cdot w_{pos}^{k}\;(\mathrm{if}\;y_{i}=1) \end{array}$$

This strategy ensures that, during each iteration, the model automatically adapts the penalty for misclassification of the minority class samples to the current data distribution, thus forcing the decision boundary to shift towards the majority class.

Considering that in the remote tower scenario, the cost of false negatives is significantly higher than that of false positives, relying solely on training weights may still fall short of safety requirements. Therefore, during the inference phase, we discard the default 0.5 probability threshold and adopt a threshold-moving strategy.

Specifically, we use the validation set to search for the optimal decision threshold $Th_{\mathrm{opt}}$, with the goal of maximizing Youden’s J statistic:

(12) $$\begin{array}{c} J(Th)=Sensitivity(Th)+Specificity(Th)-1 \end{array}$$

(13) $$\begin{array}{c} Th_{opt}=\underset{Th\in [0, 1]}{\arg\max J(Th)}\; \end{array}$$

By maximizing the J statistic, the model can maintain high specificity while prioritizing the capture of fatigue states (sensitivity), thereby mathematically enforcing the “safety-first” operational principle.

#### 3.6.3. Classification Algorithm: XGBoost

Given the challenges of limited sample size and high noise in physiological signal datasets, this study utilizes XGBoost (eXtreme Gradient Boosting) as the core classifier. XGBoost offers distinct advantages over traditional machine learning algorithms and deep neural networks for structured tabular data, including robustness through L1 and L2 regularization to prevent overfitting in small, high-dimensional datasets, and interpretability, as it automatically evaluates feature importance, thereby identifying the most critical physiological indicators for fatigue detection.

The objective function of XGBoost consists of a loss function (L) and a regularization term (Ω):

(14) $$\begin{array}{c} Obj(\theta )=\sum_{i}L(y_{i},{\hat{y}}_{i})+\sum_{k}\Omega (f_{k}) \end{array}$$

**Figure 7:**
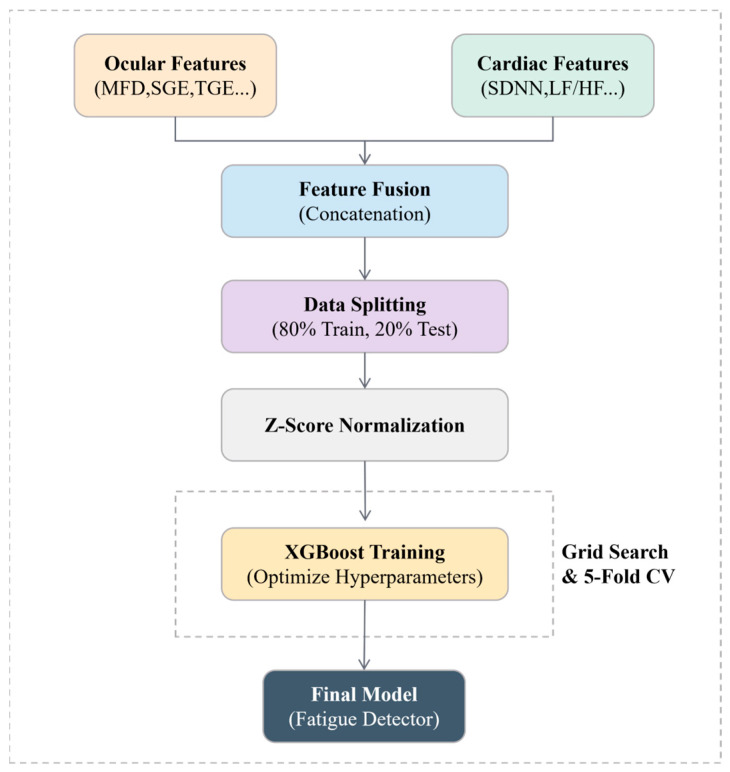
Feature fusion and model optimization framework.

During training, we used grid search combined with 5-fold cross-validation to optimize key hyperparameters (such as learning rate, maximum tree depth, and subsample ratio) to achieve the best generalization performance. The detailed training process, including feature normalization, early fusion, and hyperparameter optimization through 5-fold cross-validation, is illustrated in Figure 7.

#### 3.6.4. Deep Learning Baseline: Multi-Layer Perceptron

To explore the applicability of gradient-based deep learning models on physiological tabular data, this study constructs a standard Multi-Layer Perceptron (MLP) [53] as a baseline comparison. Unlike XGBoost, which uses a local partitioning strategy based on decision trees, the MLP relies on global dense connections to capture higher-order nonlinear interactions between features. Given the limited sample size of the physiological dataset, overly deep network architectures are prone to overfitting. The introduction of this baseline model aims to verify whether, at the current sample scale, the global feature abstraction capability of neural networks can outperform the inductive bias of ensemble tree models.

### 3.7. Personalized Calibration Strategy via Transfer Learning

Physiological signals exhibit significant inter-subject variability, causing performance degradation in general models when applied to unseen subjects. To address this cross-subject generalization issue and enhance model adaptability in real-world applications, this study proposes an individual fine-tuning strategy based on transfer learning.

The strategy involves two stages:

- General Feature Learning: a general model is trained on data from N − 1 subjects in the source domain to learn common features of fatigue across subjects, creating a robust feature extractor.
- Personalized Fine-tuning: for a new user (target domain), the model’s feature extraction network is frozen, and only the classification head is updated using a small set of user-specific calibration data (e.g., 30 samples).

This pre-training and fine-tuning approach allows for rapid adaptation to individual physiological distributions, overcoming LOSO performance bottlenecks and providing a cost-effective, high-precision personalized solution for the system’s cold-start phase.

### 3.8. Evaluation Metrics

To evaluate the fatigue detection model, key metrics derived from the confusion matrix are used, along with Area Under the Curve (AUC) as a comprehensive evaluation criterion. The definitions of the metrics are as follows, where TP, TN, FP, and FN represent True Positives, True Negatives, False Positives, and False Negatives, respectively. Accuracy, Precision, Sensitivity (Recall), Specificity, and F1-Score can be calculated as follows:

(15) $$\begin{array}{c} Accuracy=\frac{\mathrm{TP}+\mathrm{TN}}{\mathrm{TP}+\mathrm{TN}+\mathrm{FP}+\mathrm{FN}} \end{array}$$

(16) $$\begin{array}{c} Precision=\frac{TP}{TP+FP} \end{array}$$

(17) $$\begin{array}{c} Sensitivity=\frac{\mathrm{TP}}{\mathrm{TP}+\mathrm{FN}} \end{array}$$

(18) $$\begin{array}{c} Specificity=\frac{\mathrm{TN}}{\mathrm{TN}+\mathrm{FP}} \end{array}$$

(19) $$\begin{array}{c} F1=2\times \frac{Precision\times Recall}{Precision+Recall}=\frac{2TP}{2TP+FP+FN} \end{array}$$

AUC (Area Under Curve): a higher AUC indicates better classification performance across thresholds.

To assess the stability and variability of the model’s performance, we report mean ± standard deviation (SD) for all metrics across multiple cross-validation folds. This provides a clearer understanding of the model’s robustness and its ability to generalize across different data partitions.

Furthermore, to evaluate the statistical significance of performance differences among multiple models, the non-parametric Friedman test followed by post hoc Nemenyi test was employed (p<0.05).

## 4. Results

### 4.1. Experimental Design and Evaluation Settings

To evaluate the effectiveness and robustness of the proposed multimodal fatigue detection framework, a series of comprehensive experiments were conducted. All experiments were performed under a unified computational environment to ensure the fairness and comparability of the results.

#### 4.1.1. Experimental Setup

All data preprocessing, feature extraction, and model training were conducted on a high-performance workstation with an Intel Core i9-12900K CPU, 32 GB RAM, and an NVIDIA GeForce RTX 4090 GPU, which accelerated deep learning model training (e.g., transformer-based models). The software environment included Python 3.9.12, Scikit-learn 1.0.2 for data partitioning and evaluation, XGBoost 1.6.1 for classifier development, and PyTorch 1.11.0 for deep learning.

Two evaluation protocols were defined to assess model performance:

- Mixed-subject evaluation (Protocol A): A fixed random seed of 42 was used, with an 80:20 stratified split for training and testing. Five-fold cross-validation was employed within the training set for hyperparameter optimization, and model performance was evaluated on the test set.
- Cross-subject evaluation (Protocol B): A Leave-One-Subject-Out cross-validation (LOSO) procedure was performed, where each of the 36 subjects was iteratively held out as the test subject. This ensured strict test-set isolation and simulated cross-individual generalization [54].

#### 4.1.2. Hyperparameter Configuration

For hyperparameter tuning of the XGBoost model, a grid search with five-fold cross-validation was used to identify the optimal parameters. The scale_pos_weight parameter was computed to address class imbalance, adjusting the loss function to improve sensitivity to the minority fatigue class. The final configuration included a learning rate of 0.01, 500 estimators, and a maximum tree depth of eight to prevent overfitting. The subsample ratio was set to 0.7, and the column subsampling ratio (colsample_bytree) to 0.8.

For the MLP baseline, a fully connected network with two hidden layers was used. The layers had 64 and 32 units, and ReLU activation was applied. Features were standardized using Z-score normalization. The Adam optimizer was used for training, with a maximum of 500 epochs and early stopping to avoid overfitting.

### 4.2. Performance Comparison

#### 4.2.1. Comparison with Baseline Methods

To further validate the effectiveness of the proposed framework for fatigue detection, we conducted a rigorous benchmark comparison under the same data split (80% training/20% testing) against three widely used baseline classifiers, including support vector machines (SVM) [55], random forests (RF) [56], and a Multi-Layer Perceptron (MLP) [53]. As reported in Table 2, although both SVM and RF achieved an overall accuracy of 83.15%, which is comparable to that of the proposed model (83.52%), this single metric masks substantial deficiencies in the baseline models when dealing with imbalanced data.

Compared with the baseline models, the proposed XGBoost approach substantially improved sensitivity and F1-score while retaining relatively high specificity, indicating a better performance balance under imbalanced data.

**Figure 8:**
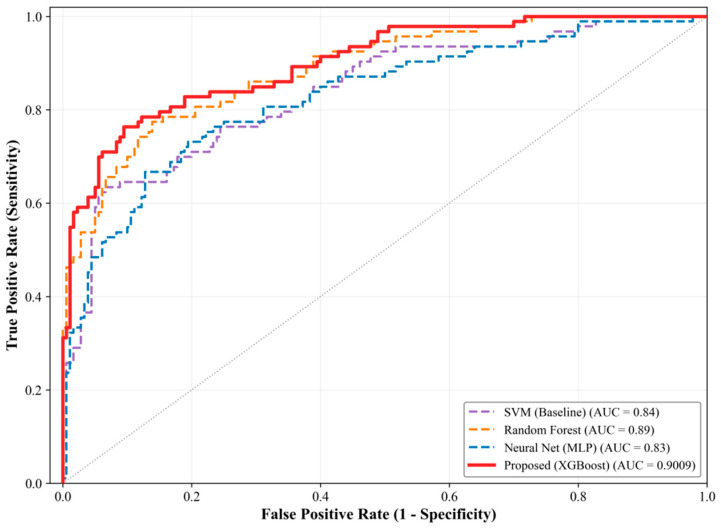
Comparison of ROC curves on the test set.

The superiority of the proposed model is further confirmed by the ROC curve comparison (Figure 8). The proposed model’s ROC-AUC of 0.9009 significantly exceeds those of Random Forest (0.8876) and SVM (0.8376), demonstrating its robustness in capturing complex and imbalanced fatigue-related patterns.

#### 4.2.2. Generalization Analysis Under LOSO

**Figure 9:**
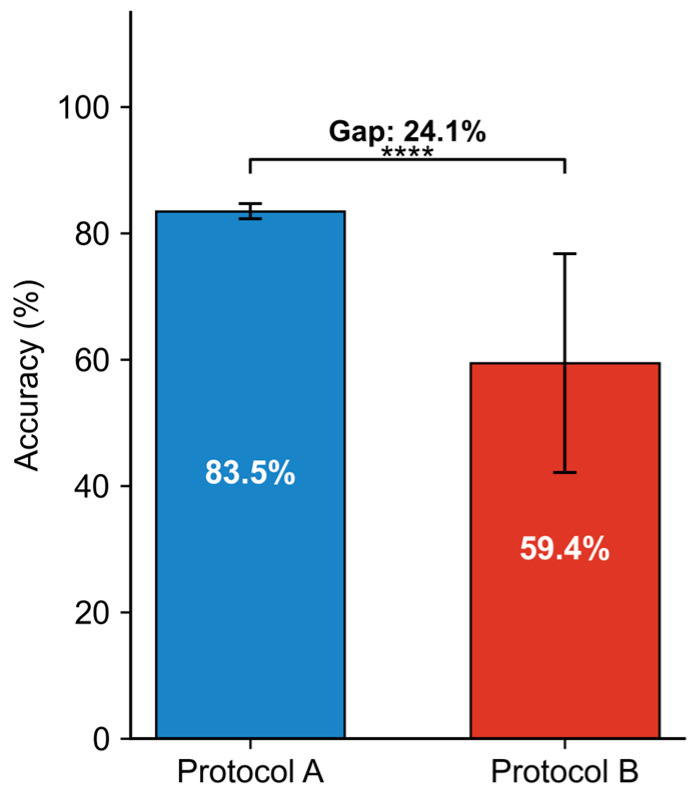
Quantification of the generalization gap between mixed-subject and cross-subject evaluations.

To investigate the impact of inter-individual variability on cross-subject transferability, we compared results under the mixed-subject protocol (Protocol A) and cross-subject protocol (Protocol B, LOSO). As shown in Figure 9, the shift from Protocol A to Protocol B led to a significant performance drop, with accuracy decreasing from 83.52% to 59.44%, resulting in a 24.1% generalization gap. This highlights the challenge of subject dependency, where generic features learned from pooled data struggle to transfer to new subjects due to inherent physiological differences. Formal statistical analysis using the Friedman test confirmed significant performance differences among models ($\chi^{2}=18.4,p<0.01$), with post hoc Nemenyi tests showing that the proposed multimodal XGBoost significantly outperforms all unimodal baselines (p<0.05).

**Figure 10:**
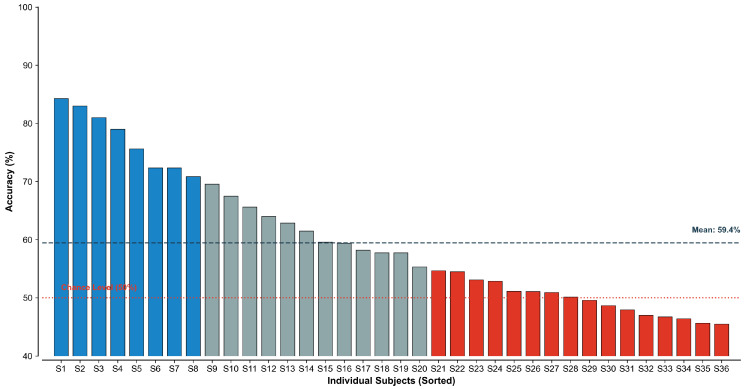
Subject-wise performance distribution demonstrating high inter-subject variability under the Leave-One-Subject-Out (LOSO) protocol.

Further inspection of individual performance under the LOSO protocol (Figure 10) reveals high variance across subjects. A small subset of participants with consistent physiological patterns achieved 70–80% accuracy, while the majority performed poorly, with some near random guessing. This variation underscores that, without personalized calibration, a generic population-trained model cannot guarantee reliable fatigue monitoring for all controllers.

### 4.3. Performance Improvement with Personalized Calibration

#### 4.3.1. Experimental Design

To address the cross-subject generalization gap (Section 4.2.2), we evaluated an active personalized calibration strategy simulating a “cold-start” scenario. Using the LOSO framework, we reserved the first 30 samples (approx. 30 min) of each test subject as a calibration set ($D_{cal}$). We applied a transfer-learning approach by fine-tuning the generic XGBoost model with $D_{cal}$ to adapt decision boundaries to individual physiological baselines.

#### 4.3.2. Results Analysis

**Figure 11:**
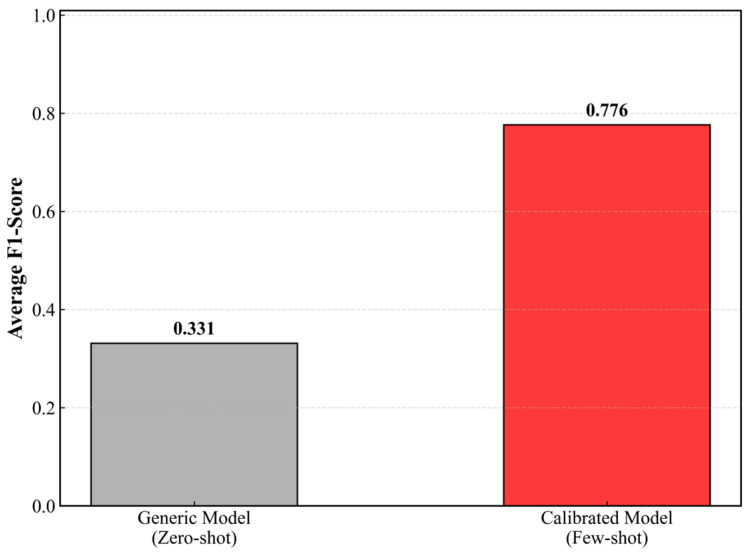
Performance comparison between the generic model (zero-shot) and the individually calibrated model (few-shot) using 30 stratified samples.

As shown in Figure 11, under this specific calibration protocol, the generic model’s baseline accuracy was 53.54%. However, incorporating just 30 subject-specific calibration samples triggered a substantial performance boost. The mean accuracy improved significantly from 53.54% to 79.01%, representing a net gain of 25.47%. Notably, the F1-score rose from 0.331 to 0.776, showing that the calibrated model achieved a better balance between precision and recall. Post-calibration, the model reached a specificity of 77.32% and sensitivity of 78.28%. A paired *t*-test confirmed that these improvements were statistically significant (p<0.001). These results demonstrate that a minimal pre-shift calibration (approx. 30 min) is sufficient to bridge the generalization gap for operational deployment. Regarding real-time feasibility, the average inference time is 24.5 ± 3.2 ms per 60-s window on a standard CPU, ensuring near-instantaneous feedback without specialized hardware.

### 4.4. Ablation Study

#### 4.4.1. Contribution of Multimodal Fusion

To evaluate the effectiveness of the multimodal fusion strategy, an ablation study was conducted using three configurations: (1) Eye-only (8-dimensional oculometric features), (2) ECG-only (4-dimensional cardiac features), and (3) Proposed Fusion (12-dimensional multimodal features). All configurations used the same XGBoost hyperparameters for a fair comparison.

**Figure 12:**
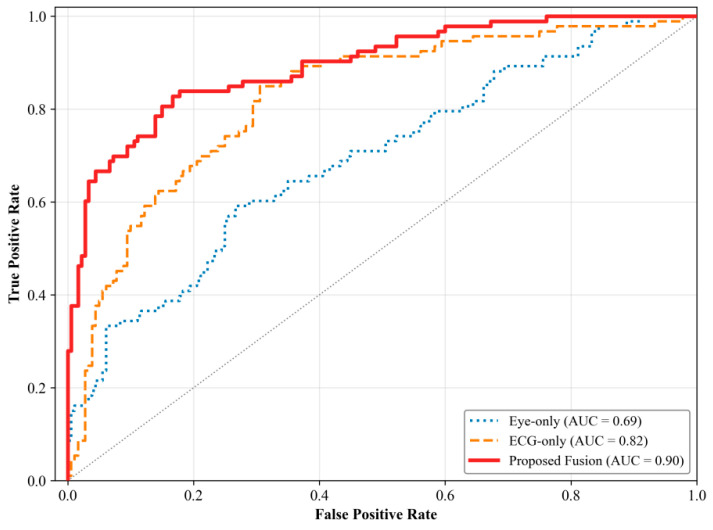
ROC curve comparison of different feature modalities.

As shown in Figure 12, the multimodal fusion (red solid curve) outperforms the single-modality models. The Eye-only model (AUC = 0.68) underperformed, indicating that visual–behavioral indicators alone are insufficient. The ECG-only model (AUC = 0.82) performed better but was still limited by its single modality. In contrast, the Proposed Fusion model achieved the highest performance (AUC = 0.90), demonstrating the superiority of combining features from both modalities.

#### 4.4.2. Impact of Imbalance Handling Strategies

To assess the effectiveness of imbalance mitigation strategies, we compared model performance under different configurations using five-fold cross-validation (Table 3). Without any treatment (baseline), the model achieved an accuracy of 84.10% ± 2.85% but had low sensitivity (62.94% ± 6.50%), indicating majority-class bias. Single strategies, such as weighted loss and SMOTE resampling, improved sensitivity to 70.92% ± 6.30% and 75.10% ± 6.20%, respectively, but each had limitations. The proposed hybrid strategy achieved the best balance, with high sensitivity (78.49% ± 6.15%) and AUC (0.9009 ± 0.024), confirming it as the optimal configuration. Furthermore, the relatively low standard deviations observed across folds (e.g., accuracy SD≈2.66%) demonstrate that the proposed framework maintains consistent performance across different data partitions, indicating robust generalization capability.

### 4.5. Feature Importance Analysis

**Figure 13:**
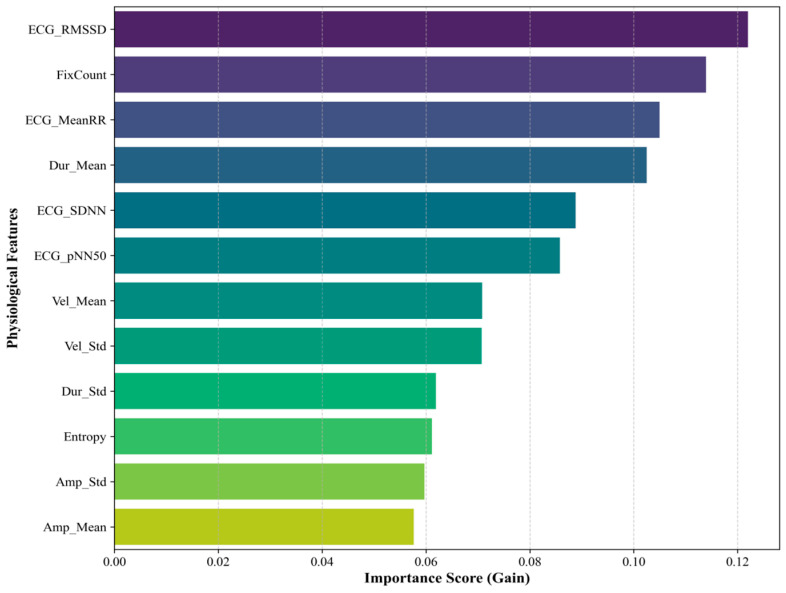
Feature importance ranking based on the XGBoost model.

To better understand the physiological patterns associated with the model’s predictions, we ranked feature importance based on the average information gain from the XGBoost model (Figure 13). The results suggest that autonomic regulation-related features, particularly ECG-derived features, may contribute to early fatigue detection. Among them, ECG_RMSSD and ECG_MeanRR showed relatively high importance, suggesting that changes in parasympathetic regulation may occur before more visible fatigue-related behaviors emerge. Eye-tracking features, such as FixCount and Dur_Mean, were also informative and may reflect reduced visual search efficiency as fatigue deepens. Together, these findings suggest that ECG and eye-tracking features provide complementary information for fatigue characterization, supporting the use of multimodal fusion in fatigue detection.

### 4.6. Multidimensional Performance Trade-Off Analysis

**Figure 14:**
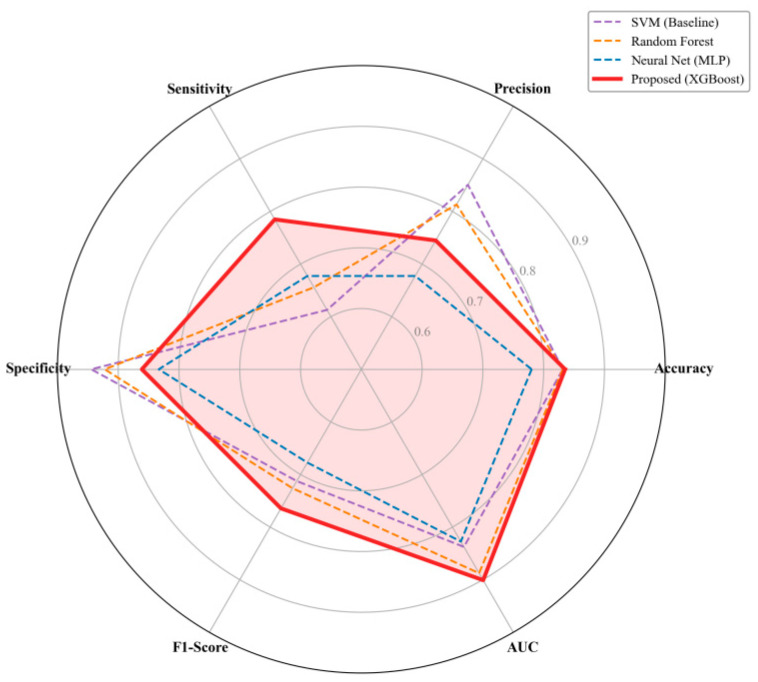
Comprehensive performance evaluation using a radar chart.

To visually assess the trade-offs among different models across multiple performance dimensions, we constructed the radar chart shown in Figure 14. The proposed XGBoost model encloses the largest area, indicating the most balanced overall performance across the evaluated metrics.

## 5. Discussion

### 5.1. Comparison with Previous Benchmarks and Safety Implications

This study proposed a non-intrusive multimodal fatigue detection framework specifically designed for remote tower (rTWR) operations. While prior studies in rTWR fatigue monitoring, such as the multimodal approaches proposed by Liang et al. [34] and the RTFnet framework by Yin et al. [35], have established robust fusion architectures, they frequently optimize for overall accuracy, which can mask the poor detection of rare fatigue events.

What distinguishes our study from this earlier research is our explicit analytical focus on severe class imbalance (16.9:1) and asymmetric safety costs. Rather than merely demonstrating that multimodal fusion works, our unique methodological contribution is the implementation of a hybrid cost-sensitive learning framework (a dynamically weighted XGBoost classifier) that mathematically enforces a “safety-first” principle.

These findings indicate that, in safety-critical remote tower operations, overall accuracy alone is insufficient; improving the detection of minority fatigue events is more important for reducing the risk of missing hazardous states.

### 5.2. Cross-Subject Generalization and Individual Variability

Although the model performed well under mixed-subject evaluation, cross-subject generalization remained constrained by substantial individual variability. This observation is consistent with prior findings in physiological fatigue research. Rim et al. [31] reported that predictive modeling based on physiological signals often shows limited generalizability across subjects. In aviation contexts, Borghini et al. [23] further noted that individual differences in autonomic baseline states, such as resting HRV, and visual scanning strategies can reduce the stability of a generic model when applied to unseen users. The high inter-subject variance observed in our LOSO evaluation (Figure 11) provides additional support for this interpretation. From an operational perspective, this limitation reduces the current readiness of the system for direct deployment in safety-critical air traffic control (ATC) environments. These findings suggest that a population-level model alone may not be sufficient for reliable rTWR fatigue monitoring. Therefore, personalized calibration should be regarded as an important step toward improving deployment readiness and mitigating the generalization gap.

### 5.3. Limitations and Future Work

Although this study has shown promising results, several limitations warrant future attention:

- Ecological validity and potential failure modes: While the simulator provides a high-fidelity environment, it cannot fully capture the stochastic nature of real-world operations, such as unexpected traffic patterns, procedural deviations, and severe weather interference. Consequently, specific failure modes may emerge in the field. For instance, sudden emergency events might induce acute stress and autonomic changes that mimic or mask fatigue, potentially leading to misclassification. Future algorithms should therefore incorporate context-aware features to distinguish stress from fatigue.
- Labeling strategy and early-warning capability: Excluding the intermediate transitional state (SP-7 = 4) oversimplifies the classification problem. Since this transitional phase is arguably the most valuable for early intervention, future work must incorporate these states to develop a three-class model (Alert–Transition–Fatigue) or explore regression-based continuous monitoring.
- Generalization and deployment constraints: The LOSO performance gap indicates the model tends to learn subject-specific patterns. Consequently, the system lacks robustness for immediate deployment on new controllers without prior calibration. Future work will explore larger-scale datasets and zero-shot learning techniques to extract invariant fatigue features.
- Real-time deployment considerations: Field deployment requires addressing latency. The system’s 60-s sliding window introduces a reporting delay, demanding continuous data streaming. Furthermore, as demonstrated in Section 4.3, a mandatory pre-shift personalized calibration is currently required to establish reliable individual baselines.

To bridge the gap between this laboratory proof-of-concept and field deployment, we propose a concrete roadmap. We acknowledge that real-world implementation faces hardware challenges, such as variable lighting affecting eye-trackers and the need for non-intrusive wearables (e.g., smart vests) to ensure comfort. To mitigate the operational risks identified above, we suggest a phased implementation: starting with an offline ‘shadow mode’ validation to calibrate decision thresholds against historical incidents, followed by a human-in-the-loop advisory pilot, and ultimately achieving full operational integration.

## 6. Conclusions

This study developed a multimodal fatigue detection framework for remote tower air traffic controllers by integrating eye-tracking and ECG/HRV features within a cost-sensitive XGBoost classifier. The proposed approach achieved strong performance under mixed-subject evaluation and improved sensitivity to fatigue events, supporting its value for safety-oriented monitoring in remote tower operations. Feature importance analysis indicated that ECG and eye-tracking features played complementary roles in characterizing fatigue. However, the marked performance decline under LOSO evaluation confirms that cross-subject variability remains a major barrier to direct deployment. Future work should therefore focus on subject-independent modeling, the inclusion of transitional fatigue states, and validation in real operational environments.

## Figures and Tables

**Table 1:** Samn–Perelli 7-point Fatigue Scale (SP-7) definitions and labeling strategy.

Category	SP-7 Score	Description	Samples (Windows)	Percentage
Alert (Negative)	1	Fully alert, wide awake	1245	36.1%
	2	Very lively, responsive, but not at peak	890	25.8%
	3	Okay, somewhat fresh	520	15.1%
Excluded (Transition)	4	A little tired, less than fresh	638	18.5%
Fatigue (Positive)	5	Moderately tired, let down	112	3.2%
	6	Extremely tired, very difficult to concentrate	35	1.0%
	7	Completely exhausted, unable to function	10	0.3%
Total	1–7	-	3450	100%

**Table 2:** Performance comparison on the test set.

Model	Accuracy	Precision	Sensitivity (Recall)	Specificity	F1-Score	AUC
SVM (Baseline)	0.8315	0.8507	0.6129	0.9444	0.7125	0.8376
Random Forest	0.8315	0.8133	0.6559	0.9222	0.7262	0.8876
Neural Net (MLP)	0.7802	0.6774	0.6774	0.8333	0.6774	0.8275
Proposed (XGBoost)	0.8352	0.7449	0.7849	0.8611	0.7644	0.9009

**Table 3:** Ablation study on imbalance strategies (average of 5-Fold CV). Data are presented as mean ± SD.

Strategy	Accuracy	AUC	Sensitivity	F1-Score
Baseline (No Treatment)	84.10% ± 2.85%	0.8657 ± 0.028	62.94% ± 6.50%	0.7014 ± 0.035
Weighted Loss Only	83.58% ± 2.70%	0.8683 ± 0.026	70.92% ± 6.30%	0.7237 ± 0.034
SMOTE Only	82.85% ± 2.75%	0.8850 ± 0.025	75.10% ± 6.20%	0.7480 ± 0.033
Hybrid (Proposed)	83.52% ± 2.66%	0.9009 ± 0.024	78.49% ± 6.15%	0.7644 ± 0.032

## Data Availability

The data generated and analyzed in this study are not publicly available due to confidentiality agreements and privacy protection concerns for the participants.

## Abbreviations

The following abbreviations are used in this manuscript:

rTWR	Remote Tower
ATC	Air Traffic Control
ECG	Electrocardiogram
HRV	Heart Rate Variability
SMOTE	Synthetic Minority Over-sampling Technique
XGBoost	eXtreme Gradient Boosting
PERCLOS	Percentage of Eyelid Closure
AUC	Area Under the Curve
LOSO	Leave-One-Subject-Out
PR-AUC	Area Under Precision-Recall Curve
ROC-AUC	Area Under Receiver Operating Characteristic Curve
